# Disparities in Hypertension Prevalence, Awareness, Treatment, and Control Among Women Living With and Without HIV in the US South

**DOI:** 10.1093/ofid/ofad642

**Published:** 2023-12-18

**Authors:** Jessica Blair, Mirjam-Colette Kempf, Jodie A Dionne, Zenoria Causey-Pruitt, Jenni M Wise, Elizabeth A Jackson, Paul Muntner, David B Hanna, Jorge R Kizer, Margaret A Fischl, Igho Ofotokun, Adaora A Adimora, Stephen J Gange, Ilene K Brill, Emily B Levitan

**Affiliations:** Department of Epidemiology, University of Alabama at Birmingham, Birmingham, Alabama, USA; Schools of Nursing, Public Health, and Medicine, University of Alabama at Birmingham, Birmingham, Alabama, USA; Division of Infectious Diseases, Department of Medicine, University of Alabama at Birmingham, Birmingham, Alabama, USA; Division of Infectious Diseases, Department of Medicine, University of Alabama at Birmingham, Birmingham, Alabama, USA; School of Nursing, University of Alabama at Birmingham, Birmingham, Alabama, USA; Division of Cardiovascular Disease, School of Medicine, University of Alabama at Birmingham, Birmingham, Alabama, USA; Department of Epidemiology, University of Alabama at Birmingham, Birmingham, Alabama, USA; Department of Epidemiology and Population Health, Albert Einstein College of Medicine, Bronx, NewYork, USA; Cardiology Section, SanFrancisco Veterans Health Care System, and Departments of Medicine, Epidemiology, and Biostatistics, University of California San Francisco, San Francisco, California, USA; Division of Infectious Diseases, Department of Medicine, Miller School of Medicine, University of Miami, Miami, Florida, USA; Division of Infectious Diseases, School of Medicine, Emory University, Atlanta, Georgia, USA; Division of Infectious Diseases, Department of Medicine, University of North Carolina at Chapel Hill, Chapel Hill, North Carolina, USA; Department of Epidemiology, Johns Hopkins Bloomberg School of Public Health, Baltimore, Maryland, USA; Department of Epidemiology, University of Alabama at Birmingham, Birmingham, Alabama, USA; Department of Epidemiology, University of Alabama at Birmingham, Birmingham, Alabama, USA

**Keywords:** antihypertensive agents, HIV, hypertension, racial and ethnic minorities, women

## Abstract

**Background:**

Hypertension-related diseases are major causes of morbidity among women living with HIV. We evaluated cross-sectional associations of race/ethnicity and HIV infection with hypertension prevalence, awareness, treatment, and control.

**Methods:**

Among women recruited into Southern sites of the Women's Interagency HIV Study (2013–2015), hypertension was defined as (1) systolic blood pressure ≥140 mm Hg or diastolic blood pressure ≥90 mm Hg according to clinical guidelines when data were collected, (2) self-report of hypertension, or (3) use of antihypertensive medication. Awareness was defined as self-report of hypertension, and treatment was self-report of any antihypertensive medication use. Blood pressure control was defined as <140/90 mm Hg at baseline. Prevalence ratios for each hypertension outcome were estimated through Poisson regression models with robust variance estimators adjusted for sociodemographic, behavioral, and clinical risk factors.

**Results:**

Among 712 women, 56% had hypertension and 83% were aware of their diagnosis. Of those aware, 83% were using antihypertensive medication, and 63% of those treated had controlled hypertension. In adjusted analyses, non-Hispanic White and Hispanic women had 31% and 48% lower prevalence of hypertension than non-Hispanic Black women, respectively. Women living with HIV who had hypertension were 19% (*P* = .04) more likely to be taking antihypertension medication when compared with women living without HIV.

**Conclusions:**

In this study population of women living with and without HIV in the US South, the prevalence of hypertension was lowest among Hispanic women and highest among non-Hispanic Black women. Despite similar hypertension prevalence, women living with HIV were more likely to be taking antihypertensive medication when compared with women living without HIV.

## Teaser

Black women had a higher prevalence of hypertension when compared with White and Hispanic women. Additionally, treatment for hypertension is higher in women with HIV than women without HIV, which may result from greater access and engagement with medical care.

Nearly 50% of US adults aged >18 years have hypertension according to the 2017 definition endorsed by the American College of Cardiology and the American Heart Association (≥130/80 mm Hg) [1, 2]. Hypertension was the highest-ranked cause of death globally in 2019, accounting for 20% of deaths among women [3]. Blood pressure control can improve quality of life, reduce cardiovascular disease (CVD) events, and increase life expectancy [3–5]. However, only 53% of US women with hypertension have controlled blood pressure [6]. Of those with uncontrolled hypertension, 50% use antihypertensive medication [1].

Non-Hispanic (NH) Black women have disproportionally high rates of hypertension mortality within the US as compared with NH White and Hispanic women [1, 7–9]. The prevalence of hypertension in NH Black, NH White, and Hispanic US women ≥20 years old was 58%, 41%, and 41%, respectively, in 2015 to 2018 [8]. NH Black adults were more likely to be aware and treated for hypertension than NH White adults in 2018 [9]. Despite higher antihypertensive treatment rates among those with hypertension, Black individuals had the highest prevalence of uncontrolled hypertension among the 3 groups [1, 10]. These disparities in hypertension outcomes may extend to persons living with HIV, who often experience intersectional stigma and discrimination [11]; few studies have evaluated the hypertension treatment cascade among women living with HIV (WLWH).

Because of the contribution of hypertension-related diseases to morbidity, management of hypertension has emerged as a priority among WLWH, who have a lifetime CVD risk of 44% [12]. Similar to the geographic distribution of hypertension within the United States, people in the South experience the greatest proportion of new HIV diagnoses when compared with other regions [13]. Several Southern states, including Alabama, Georgia, Florida, Mississippi, and North Carolina, have a mortality rate for persons living with HIV that is 3 times higher than that of non-Southern states [13]. Persons living with HIV have a higher prevalence of hypertension than those living without HIV [14–16]. However, when compared with demographically similar women living without HIV (WLWOH), WLWH typically have increased access to health care and more regular interaction with physicians, which could lead to improved hypertension awareness, treatment, and control [17, 18].

The goal of this study was to evaluate cross-sectional associations between race/ethnicity and prevalence, awareness, treatment, and control of hypertension among women in the South participating in the Women's Interagency HIV Study (WIHS). We also evaluated associations between HIV and prevalence, awareness, treatment, and control of hypertension. Our study was designed to test 2 hypotheses: (1) prevalence, awareness, and treatment of hypertension would be higher and control of hypertension would be lower in NH Black women vs other groups, and (2) WLWH would have a higher prevalence as well as higher awareness, treatment, and control of hypertension in comparison with sociodemographically similar WLWOH.

## METHODS

### Population

WIHS was created by the US National Institutes of Health in 1993 to investigate HIV among women and has been described in detail [19]. WLWH and WLWOH sociodemographically matched to WLWH participated in twice-yearly study visits where behavioral and clinical data were collected. WIHS merged with the Multicenter AIDS Cohort Study (MACS) in 2019 to create the MACS/WIHS Combined Cohort Study (MWCCS), which has also been described [20].

The current study included cis-gender women who were recruited into WIHS between 2013 and 2015 as part of the Southern expansion that enrolled women in the following cities: Atlanta, Georgia; Miami, Florida; Birmingham, Alabama/Jackson, Mississippi; and Chapel Hill, North Carolina (n = 845). Women from non-Southern sites were enrolled in the WIHS in different recruitment waves that spanned from 1993 to 2015. Women enrolled in Southern sites were recruited only in the last recruitment wave; therefore, analyses were limited to women at Southern sites to help control for major secular trends in hypertension outcomes. Inclusion criteria for WLWH were documentation of a reactive HIV serology, prescription of highly active antiretroviral therapy (ART), or prescription of non–highly active ART during pregnancy [19]. Inclusion criteria for WLWOH were at least 1 of the following high-risk exposures within the last 5 years: sexually transmitted infection, injection drug use, sex with a man who had HIV infection, or sex with multiple men [19]. For the current analyses, participants were excluded if they were missing data needed to determine prevalence, awareness, treatment, and control of hypertension, as were participants missing data on race/ethnicity, HIV status, and covariates.

WIHS participants provided written informed consent, and participating sites received institutional review board approval before enrolling participants. This analysis received approval by the MWCCS executive committee and University of Alabama at Birmingham institutional review board. Data necessary to replicate these analyses are available from MWCCS (https://statepi.jhsph.edu/mwccs/work-with-us/, mwccs@jhu.edu). The lead author (J. B.) had full access to all data in her study and takes full responsibility for their integrity and analysis.

### Outcomes

Blood pressure was measured with an automated monitor (Dinamap Procare Series; GE Medical Systems) for standardization. Proper cuff size was determined by measuring the arm circumference of the midpoint between the shoulder and the elbow. Participants were seated with both feet flat on the floor for 5 minutes before the first reading. Three blood pressure readings were obtained with 1-minute intervals in between. Blood pressure medication use was assessed through a combination of self-report, review of pill bottles, and medication lists. The outcomes of interest were prevalence, awareness, treatment, and control of hypertension at the baseline study visit. Participants were considered to have hypertension if their mean systolic or diastolic blood pressure was ≥140/90 mm Hg at the baseline visit or they reported a medical history of hypertension or use of antihypertensive medication. We chose this definition because the recommended threshold to define hypertension was ≥140/90 mm Hg when baseline data were collected [1]; the diagnostic criterion for hypertension diagnosis was revised to ≥130/80 mm Hg in 2017 [21]. Awareness was defined by participants with hypertension knowing the status of their hypertension diagnosis. Participants were asked, “Have you ever had high blood pressure or hypertension that required medical care?” For those aware of hypertension, participants were considered to be undergoing treatment for it if they were currently taking antihypertensive medication, which was self-reported and verified through review of medical records with participants. Among participants treated for hypertension, control of hypertension was defined as mean systolic and diastolic blood pressure <140/90 mm Hg.

### Main Comparisons

Our primary comparisons were among women of different race/ethnicity. Hispanic ethnicity was recorded for participants who gave an affirmative response to the question “Are you of Hispanic (Spanish) or Latina origin?” Race was self-reported as White, Black, Asian, Pacific Islander, American Indian/Alaskan, and other. Due to the small sample sizes of some groups, we categorized race/ethnicity as Hispanic (of any race), NH Black, and NH White. We conducted only descriptive analyses for women who reported race/ethnicity identities other than Hispanic (of any race), NH Black, and NH White because of small sample sizes. We also compared women living with and without HIV at baseline visit. A sensitivity analysis was explored by HIV status, defined as negative, suppressed viral load (<20 copies/mL), and unsuppressed viral load.

### Covariates

All covariates were captured at baseline visit. Sociodemographic, behavioral, and clinical covariates were selected by known or hypothesized associations with race/ethnicity, HIV, and hypertension outcomes that could lead to confounding. The sociodemographic factors that we considered were age, highest education level, and health insurance, which were all captured through questionnaires. Education was categorized as less than high school, high school graduate, some college, and college graduate or higher. Health insurance was categorized as uninsured, Medicaid only, AIDS Drug Assistance Program only, and other, which includes >1 type of insurance. The behavioral factors were smoking status (never, current, or former), current alcohol use, and substance use. Alcohol use was categorized as none, moderate (1–7 drinks/wk), and heavy (>7 drinks/wk) according to the National Institute on Alcohol Abuse and Alcoholism guidelines for women [22]. Substance use was categorized as ever use of intravenous drugs, nonintravenous drugs (crack, cocaine, heroin, or methadone) other than marijuana, marijuana only, and none. The clinical risk factors in the analyses were body mass index (kg/m^2^; underweight/normal, <25; overweight, 25 to <30; obesity, ≥30), diabetes, estimated glomerular filtration rate (based on Chronic Kidney Disease Epidemiology Collaboration definition with race) [23], hepatic fibrosis [24], aspartate aminotransferase/platelet ratio, hepatitis C virus infection status, depressive symptoms per the Center for Epidemiologic Studies–Depression scale, and history of CVD. Diabetes was defined as fasting glucose ≥126 mg/dL, hemoglobin A_1c_ ≥6.5%, confirmed self-report diagnosis, or ever self-reported antidiabetic medication. A participant was considered to have depressive symptoms if the Center for Epidemiologic Studies–Depression score was ≥16 [25]. Self-reported history of CVD was defined as heart attack, stent, stroke, chest pain, or hospitalization for heart condition. HIV-specific characteristics consisted of duration of ART in years, CD4 count, current ART, and history of AIDS diagnosis (yes/no). ART usage at baseline was categorized as none, regimen including integrase inhibitors, and regimen not including integrase inhibitors due to known CVD risk with integrase inhibitors [26].

### Statistical Methods

We first described baseline characteristics of study participants by race/ethnicity and HIV status with mean (SD) or number (percentage). This cross-sectional study estimated prevalence ratios (PRs) for each hypertension outcome through Poisson regression models with robust variance estimators [27]. The crude PR model for our main comparison included only race/ethnicity (model 1). The fully adjusted model (model 2) comprised sociodemographic factors, behavioral factors, and clinical risk factors. HIV-related variables (current ART usage, duration of ART, and history of AIDS diagnosis) were included by creating interaction terms with HIV status. The crude PR model for our secondary comparison consisted only of HIV status (model 1). The fully adjusted model (model 2) added sociodemographic factors, behavioral factors, and clinical risk factors. The fully adjusted model did not include duration of ART, AIDS diagnosis, or ART regimen because these variables applied only to persons living with HIV.

We conducted a sensitivity analysis using the 2017 American College of Cardiology/American Heart Association definition of hypertension (≥130/80 mm Hg) and hypertension control (<130/80 mm Hg) [21]. We conducted the following exploratory analyses: multiple imputation by chained equations to account for missing data for covariates; HIV status further categorized as negative, suppressed viral load, and unsuppressed viral load; comparison of race/ethnicity among WLHIV; and race categorized as Black and non-Black. The following covariates had missing data: health insurance, alcohol use, body mass index, estimated glomerular filtration rate, hepatic fibrosis, aspartate aminotransferase/platelet ratio, hepatitis C virus infection status, depression, duration of ART, and CD4 count.

## RESULTS

In total, 845 women were recruited into the Southern sites. We excluded 2 participants who transferred into the Southern sites. In the primary analyses, participants who reported Asian/Pacific Islander, American Indian/Alaskan, and other race/ethnicity were excluded because the number of participants was too small to draw conclusions (n = 15). The hypertension outcomes for these individuals are described in Supplementary Figure 1. Participants missing blood pressure measurements (n = 3) and data on covariates (n = 113) were also excluded (Supplementary Figure 2). See Supplementary Table 1 for characteristics of those excluded.

Our final sample included 712 women, of whom 602 (84%) were NH Black, 70 (10%) were NH White, and 40 (6%) were Hispanic. There were 493 (69%) WLHIV. Hispanic WLWH had the highest prevalence of history of AIDS at the time of study entry (13%) when compared with NH Black and NH White women (7% and 9%, respectively; Table 1). NH White and Hispanic women each had a lower prevalence of obesity and diabetes when compared with NH Black women. Nearly three-quarters of WLWH had suppressed viral loads; 62% were taking non–integrase inhibitors; and 40% had only Medicaid, as opposed to 47% of WLWOH who were uninsured. See Table 1 for the summary of the remaining characteristics.

**Table 1.** Characteristics of Study Population for Women Enrolled at Southern Sites of the Women's Interagency HIV Study (N = 712)

	Overall (N = 712)	NH Black (n = 602)	NH White (n = 70)	Hispanic (n = 40)	WLWH (n = 493)	WLWOH (n = 219)
Baseline age, y	43.2 ± 9.4	43.3 ± 9.5	44.0 ± 9.1	40.8 ± 9.1	43.8 ± 9.2	42.0 ± 9.7
Race/ethnicity						
NH Black	602 (84.6)	…	…	…	417 (84.6)	185 (84.5)
NH White	70 (9.8)	…	…	…	49 (9.9)	21 (9.6)
Hispanic	40 (5.6)	…	…	…	27 (5.5)	13 (5.9)
Education						
Less than high school	214 (30.1)	181 (30.1)	17 (24.3)	16 (40.0)	148 (30.0)	66 (30.1)
High school graduate	222 (31.2)	179 (29.7)	26 (37.1)	17 (42.5)	166 (33.7)	56 (25.6)
Some college	226 (31.7)	197 (32.7)	23 (32.9)	6 (15.0)	145 (29.4)	81 (37.0)
College graduate or higher	50 (7.0)	45 (7.5)	4 (5.7)	1 (2.5)	34 (6.9)	16 (7.3)
Health insurance						
Uninsured	166 (23.3)	132 (21.9)	20 (28.6)	14 (35.0)	63 (12.8)	103 (47.0)
Medicaid only	261 (36.7)	226 (37.5)	24 (34.3)	11 (27.5)	198 (40.2)	63 (28.8)
ADAP only	107 (15.0)	84 (14.0)	11 (15.7)	12 (30.0)	107 (21.7)	0 (0.0)
Other^a^	178 (25.0)	160 (26.6)	15 (21.4)	3 (7.5)	125 (25.4)	53 (24.2)
Smoking status						
Never	289 (40.6)	250 (41.5)	17 (24.3)	22 (55.0)	217 (44.0)	72 (32.9)
Former	93 (13.1)	76 (12.6)	14 (20.0)	3 (7.5)	71 (14.4)	22 (10.1)
Current	330 (46.4)	276 (45.9)	39 (55.7)	15 (37.5)	205 (41.6)	125 (57.1)
Alcohol use,^b^ %						
None	336 (47.2)	278 (46.2)	34 (48.6)	24 (60.0)	245 (49.7)	91 (41.6)
Moderate	251 (35.3)	219 (36.4)	22 (31.4)	10 (25.0)	181 (36.7)	70 (32.0)
Heavy	125 (17.6)	105 (17.4)	14 (20.0)	6 (15.0)	67 (13.6)	58 (26.5)
Substance use						
None	233 (32.7)	197 (32.7)	13 (18.6)	23 (57.5)	178 (36.1)	55 (25.1)
Marijuana only	117 (16.4)	99 (16.5)	14 (20.0)	4 (10.0)	74 (15.0)	43 (19.6)
Nonintravenous drug use	316 (44.4)	278 (46.2)	29 (41.4)	9 (22.5)	210 (42.6)	106 (48.4)
Intravenous drug use	46 (6.5)	28 (4.7)	14 (20.0)	4 (10.0)	31 (6.3)	15 (6.9)
Body mass index,^c^ %						
Underweight/normal	123 (17.3)	95 (15.8)	18 (25.7)	10 (25.0)	88 (17.9)	35 (16.0)
Overweight	163 (22.9)	130 (21.6)	18 (25.7)	15 (37.5)	122 (24.8)	41 (18.7)
Obese	426 (59.8)	377 (62.6)	34 (48.6)	15 (37.5)	283 (57.4)	143 (65.3)
History of CVD,^d^ %	64 (9.0)	56 (9.3)	5 (7.1)	3 (7.5)	41 (8.3)	23 (10.5)
Diabetes,^e^ %	84 (11.8)	76 (12.6)	5 (7.1)	3 (7.5)	56 (11.4)	28 (12.8)
eGFR	100.8 ± 23.7	102.3 ± 23.8	86.4 ± 22.2	103.4 ± 16.8	98.5 ± 24.5	106.1 ± 21.1
Hepatic fibrosis	1.06 ± 1.0	1.1 ± 1.0	1.1 ± 0.7	1.0 ± 0.6	1.1 ± 0.8	0.98 ± 1.2
APRI	0.3 ± 0.4	0.3 ± 0.4	0.3 ± 0.4	0.3 ± 0.2	0.3 ± 0.3	0.3 ± 0.6
Hepatitis C	84 (11.8)	62 (10.3)	19 (27.1)	3 (7.5)	56 (11.4)	28 (12.8)
Depressive symptoms^f^	327 (45.9)	272 (45.2)	37 (52.9)	18 (45.0)	217 (44.0)	110 (50.2)
HIV status						
Negative	219 (30.8)	185 (30.7)	21 (30.0)	13 (32.5)	…	219 (100.0)
Suppressed^g^	363 (51.0)	308 (51.2)	39 (55.7)	16 (40.0)	363 (73.6)	…
Unsuppressed	130 (18.3)	109 (18.1)	10 (14.3)	11 (27.5)	130 (26.4)	…
Current ART usage						
None	28 (5.7)	25 (6.0)	2 (4.1)	1 (3.7)	28 (5.7)	…
INSTIs	162 (32.9)	130 (31.2)	22 (44.9)	10 (37.0)	162 (32.9)	…
Non-INSTIs	303 (61.5)	262 (62.8)	25 (51.0)	16 (59.3)	303 (61.5)	…
Duration of ART, y	4.1 ± 2.7	4.1 ± 2.7	3.6 ± 2.4	4.3 ± 3.1	4.1 ± 2.7	…
CD4 count, cells/μL	752.5 ± 414.6	746.2 ± 405.6	835.2 ± 462.7	702.3 ± 450.9	601.9 ± 319.4	1091.6 ± 404.3
AIDS diagnosis	54 (7.6)	43 (7.1)	6 (8.6)	5 (12.5)	54 (11.0)	…

Data are presented as mean ± SD or No. (%).

Abbreviations: ADAP, AIDS Drug Assistance Program; APRI, aspartate aminotransferase/platelet ratio; ART, antiretroviral therapy; CVD, cardiovascular disease; eGFR, estimated glomerular filtration rate; INSTI, integrase inhibitor; NH, non-Hispanic; WLWH, women living with HIV; WLWOH, women living without HIV.

^a^Other includes private, Medicare, combination of insurance, and other insurance.

^b^Rank based on none, moderate (1–7 drinks/wk), and heavy (>7 drinks/wk) according to National Institute on Alcohol Abuse and Alcoholism guidelines for women.

^c^Body mass index (kg/m^2^) defined as underweight/normal (<25), overweight (25 to <30), and obese (≥30).

^d^History of CVD includes, myocardial infarction, hospitalization for congestive heart failure, stroke, transient ischemic attack, hospitalization for angina, or surgery on heart vessels.

^e^Diabetes defined as fasting glucose ≥126 mg/dL, hemoglobin A_1c_ ≥6.5%, confirmed self-report diagnosis, or ever self-reported antidiabetic medication.

^f^Depressive symptoms defined Center for Epidemiologic Studies–Depression score ≥16.

^g^Cutoff for viral suppression was <20 copies/mL.

**Figure 1.**
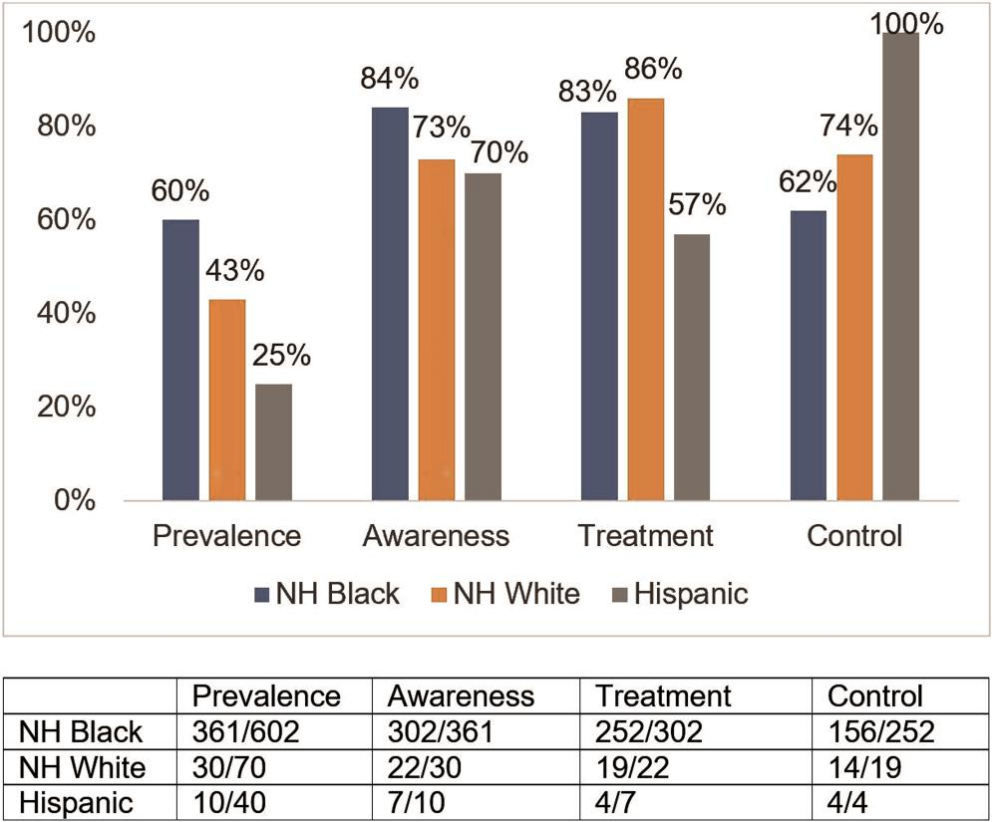
Proportions of hypertension outcomes by race/ethnicity for women enrolled at Southern sites of the Women's Interagency HIV Study (N = 712). NH, non-Hispanic.

Within our sample, 401 (56%) women had hypertension, and 331 (83%) with hypertension were aware of their diagnosis. Of those aware of their hypertension status, 83% were currently taking antihypertensive medication, and 63% of women who were treated for hypertension had it under control. The prevalence of hypertension was greater for NH Black women (60%) than for NH White and Hispanic women (43% and 25%, respectively; Figure 1). After adjustment for sociodemographic factors, behavioral factors, and clinical risk factors, NH White and Hispanic women had a significantly lower prevalence of hypertension than NH Black women (PR, 0.69 [95% CI, .54–.90]; PR, 0.52 [95% CI, .32–.85]; *P* < .0001; Table 2). Associations were similar in the sensitivity analysis with hypertension defined as ≥130/80 mm Hg (Supplementary Table 2). Sensitivity analysis with multiple imputation by chained equations for missing covariates and race categorized as Black/non-Black showed similar results as Table 2 (Supplementary Tables 3 and 4). Among WLWH, NH White and Hispanic women had a lower prevalence of hypertension than NH Black women (PR, 0.75 [95% CI, .56–.99]; PR, 0.56 [95% CI, .32–.98]; *P* = .0058; Supplementary Table 5).

**Table 2.** Unadjusted and Adjusted Prevalence Ratios and 95% CIs for the Association Between Hypertension Outcomes and Race/Ethnicity for Women Enrolled at Southern Sites of the Women's Interagency HIV Study

	Prevalence Ratio (95% CI)			
	Model 1^a^	*P* value^b^	Model 2^c^	*P* value^b^
Prevalence (n = 712)		**<**.**0001**		**<**.**0001**
NH Black	1 [Reference]		1 [Reference]	
NH White	0.71 (.54–.94)		0.69 (.54–.90)	
Hispanic	0.42 (.24–.72)		0.52 (.32–.85)	
Awareness (n = 401)		.3273		.1097
NH Black	1 [Reference]		1 [Reference]	
NH White	0.88 (.70–1.09)		0.83 (.68–1.02)	
Hispanic	0.84 (.56–1.26)		0.84 (.59–1.17)	
Treatment (n = 331)		.5253		.3477
NH Black	1 [Reference]		1 [Reference]	
NH White	1.02 (.83–1.25)		0.95 (.79–1.16)	
Hispanic	0.71 (.37–1.36)		0.72 (.43–1.19)	
Control (n = 275)		.0752		.0618
NH Black	1 [Reference]		1 [Reference]	
NH White	1.19 (.89–1.58)		1.19 (.86–1.65)	
Hispanic	1.62 (1.47–1.78)		2.12 (1.52–2.96)	

Abbreviation: NH, non-Hispanic.

^a^Race/ethnicity only (unadjusted).

^b^Type 3 significant *P* value indicated that at least 1 group is different from another. Bold type indicates significance.

^c^Model 1 + all covariates (fully adjusted). Fully adjusted Poisson model includes the following variables: age, education, health insurance, smoking, alcohol, substance use, body mass index, history of cardiovascular disease, diabetes, estimated glomerular filtration rate, hepatic fibrosis, aspartate aminotransferase/platelet ratio, hepatitis C, depressive symptoms, CD4 count, HIV, and interactions between HIV and antiretroviral therapy, duration of antiretroviral therapy, and AIDS.

The proportion of awareness was higher among NH Black women (84%) than among NH White and Hispanic women (73% and 70%, respectively). The proportion of controlled hypertension was lower for NH Black women (62%) vs NH White and Hispanic women (74% and 100%; Figure 1), but hypertension control among Hispanic women was significantly higher than among Black women in unadjusted and adjusted analyses, although this was based on a small number of Hispanic women (Table 2).

**Figure 2.**
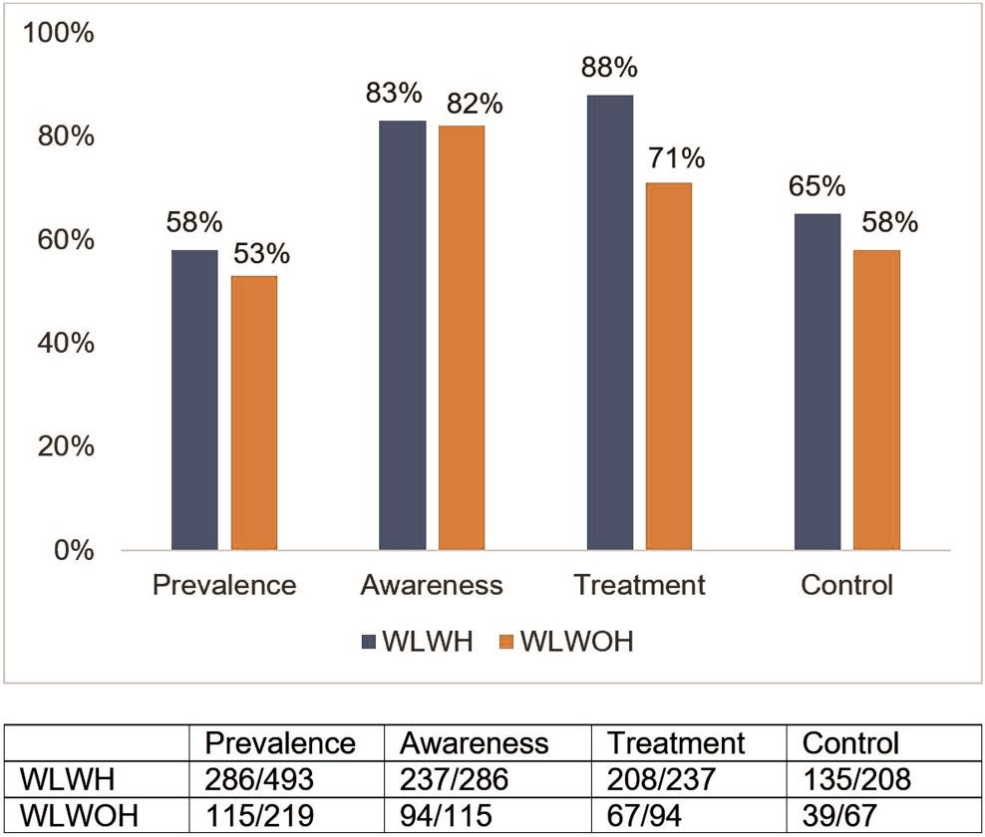
Proportions of hypertension outcomes by HIV status for women enrolled at Southern sites of the Women's Interagency HIV Study (N = 712). WLWH, women living with HIV; WLWOH, women living without HIV.

Figure 2 shows the proportions of hypertension, awareness, treatment, and control by HIV status. Numerically, the proportions of all hypertension outcomes were higher among WLWH than WLWOH before confounder adjustment. For example, WLWH had higher proportions of antihypertension treatment when compared with WLWOH (88% and 71%, respectively), and this association persisted in adjusted analyses (PR, 1.19 [95% CI, 1.01–1.40]; *P* = .0353; Table 3). WLWH also had a higher prevalence of hypertension control (65%) vs WLWOH (58%), although this difference was not statistically significant in adjusted analyses. Further unadjusted exploratory analyses showed that WLWOH were less likely to be taking antihypertensive medication when compared with women with viral suppression (PR, 0.81 [95% CI, .70–.93]; *P* = .0098; Supplementary Table 6). Multiple imputation by chained equations for missing covariates and hypertension defined as ≥130/80 mm Hg showed similar results as Table 3 (Supplementary Tables 7 and 8). See Supplementary Table 9 for associations between covariates and hypertension outcomes.

**Table 3.** Unadjusted and Adjusted Prevalence Ratios and 95% CIs for the Association Between Hypertension Outcomes and HIV Status for Women Enrolled at Southern Sites of the Women's Interagency HIV Study

	Prevalence Ratio (95% CI)			
	Model 1^a^	*P* Value	Model 2^b^	*P* Value
Prevalence (n = 712)		.1744		.0592
WLWOH	1 [Reference]		1 [Reference]	
WLWH	1.10 (0.95–1.28)		1.18 (0.99–1.40)	
Awareness (n = 401)		.7900		.4872
WLWOH	1 [Reference]		1 [Reference]	
WLWH	1.01 (0.92–1.12)		1.05 (0.92–1.19)	
Treatment (n = 331)		.**0065**		.**0353**
WLWOH	1 [Reference]		1 [Reference]	
WLWH	1.21 (1.05–1.41)		1.19 (1.01–1.40)	
Control (n = 275)		.3321		.4931
WLWOH	1 [Reference]		1 [Reference]	
WLWH	1.12 (0.89–1.40)		1.10 (0.83–1.47)	

Bold type indicates significance.

Abbreviations: WLWH, women living with HIV; WLWOH, women living without HIV.

^a^HIV status only (unadjusted).

^b^Model 1 + all covariates (fully adjusted). Fully adjusted Poisson model includes the following variables: race/ethnicity, age, education, health insurance, smoking, alcohol, substance use, body mass index, history of cardiovascular disease, diabetes, estimated glomerular filtration rate, hepatic fibrosis, aspartate aminotransferase/platelet ratio, hepatitis C, depressive symptoms, and CD4 count.

## DISCUSSION

In the current study of women enrolled in WIHS sites in the South, Hispanic women had 48% and NH White women had 30% lower prevalence of hypertension when compared with NH Black women after adjusting for covariates. Additionally, we found that Hispanic women who were prescribed antihypertensive medication were more likely to have controlled hypertension than NH Black women. These results are similar to findings from the National Health and Nutrition Examination Survey, which is representative of the noninstitutionalized civilian population of the United States [28]. Among participants in the US AIDS Drug Assistance Program for prescription assistance among persons living with HIV in WIHS, Black women were more likely to use antihypertension medication than NH White women and other ethnicities [29]. This suggests that the same contextual factors that lead to greater hypertension prevalence and less control among Black Americans generally are also drivers of hypertension disparities among WLWH and sociodemographically matched WLWOH.

WLWH were 19% more likely to be taking antihypertensive medication when compared with sociodemographically matched WLWOH. The overall adjusted PRs for each hypertension outcome were higher among WLWH than WLWOH but nonsignificant, with relatively wide confidence intervals. A 2021 study examining the association between hypertension and HIV infection among women in the WIHS cohort showed similar nonsignificant results [30]. In contrast, a global meta-analysis of cross-sectional studies found that persons living with HIV had a higher prevalence of hypertension than persons living without HIV [31, 32]. Hypertension and suboptimal treatment of hypertension may share risk factors with HIV, such as poverty, racism, and lack of access to health care. Associations between HIV infection and hypertension outcomes in broadly defined study populations may be in part due to residual confounding from sociodemographic and behavioral risk factors for HIV that overlap with risk factors for hypertension. Although our sample size is relatively small, women in WIHS have similar demographic and behavioral factors, regardless of HIV status. This may explain the less dramatic differences in hypertension outcomes between WLWH and WLWOH in this population when compared with other study populations.

Clinical implications of controlled hypertension include a lower risk of the following conditions as compared with uncontrolled hypertension: CVD, congestive heart failure, stroke, myocardial infarction, renal disease, and cognitive dysfunction [33, 34]. When compared with lower adherence, prolonged adherence of antihypertension medication can lower risk of adverse cardiovascular events by 38% [35]. When compared with WLWOH, WLWH have increased risk of hypertension-related events, such as myocardial infarction, coronary revascularization, stroke, and heart failure [36, 37]. Accordingly, screening for and treatment of hypertension are imperative for reducing hypertension-related morbidity and mortality among WLWH. Research on adherence to antihypertensive medications, lifestyle interventions to manage blood pressure, optimal antihypertensive medication regimens, and integration of blood pressure management into other types of health care for WLWH and women at risk for HIV can help to address the disparities identified in this study and similar studies.

This analysis addresses a gap in literature on the hypertension cascade of prevalence, awareness, treatment, and control among WLHIV and women at risk of HIV. The study had several strengths, such as standardized measurements across sites and generalizability of results among WLWH and sociodemographically matched women in the Southern United States. We recognize that this study is not without its limitations. Due to the nature of self-report, bias can occur in participants' answers to questions that are not verified by medical records or laboratory results. Since this is a cross-sectional analysis, we could not evaluate lifestyle interventions (eg, low-salt diet or weight loss) that may be used as first-line therapy for hypertension before initiating antihypertensive medications. WIHS did not collect information to determine adherence of antihypertensive medication for this study population at baseline. Participants who were missing data to determine race/ethnicity and covariates were excluded, which limited our sample size. Few women who were identified as races other than Black or White were included, preventing us from conducting in-depth analyses among these women. Additionally, guidelines for diagnosis of hypertension require that blood pressure be elevated across several measurement occasions; we relied on blood pressure measured at a single baseline study visit, which may have led to under- or overestimation of the prevalence of hypertension.

The patterns of hypertension prevalence, awareness, treatment, and control by race/ethnicity in our study of WLWH and women vulnerable to HIV acquisition generally aligned with prior studies in the US general population. We did not find statistically significant differences between HIV status and hypertension prevalence, awareness, or control, possibly because participants in WIHS living with and without HIV are sociodemographically matched and because of the relatively small sample size. WLWH were more likely to be treated for hypertension than WLWOH, which may reflect better access and more frequent care associated with HIV treatment. The high prevalence of hypertension and substantial proportion of individuals whose hypertension was not controlled, particularly among Black women, indicates a need for continuing efforts to diagnosis and effectively treat hypertension in this population.

## Supplementary Material

ofad642_Supplementary_Data

## Supplementary Data

Supplementary materials are available at *Open Forum Infectious Diseases* online. Consisting of data provided by the authors to benefit the reader, the posted materials are not copyedited and are the sole responsibility of the authors, so questions or comments should be addressed to the corresponding author.

## Notes

***Acknowledgments.*** We acknowledge the principal investigators of each WIHS site: Mirjam-Colette Kempf and Deborah Konkle-Parker, University of Alabama–Mississippi; Ighovwerha Ofotokun and Gina Wingood, Atlanta; Margaret Fischl and Lisa Metsch, Miami; Adaora Adimora, University of North Carolina at Chapel Hill; and Stephen Gange and Elizabeth Golub, WIHS Data Management and Analysis Center. We thank the patients, coinvestigators, and research staff from all participating sites of the WIHS.

***Data collection*.** Data in this article were collected by the WIHS, now the MWCCS. The MWCCS is funded primarily by the National Heart, Lung, and Blood Institute, with cofunding from the Eunice Kennedy Shriver National Institute of Child Health and Human Development, National Human Genome Research Institute, National Institute on Aging, National Institute of Dental and Craniofacial Research, National Institute of Allergy and Infectious Diseases, National Institute of Neurological Disorders and Stroke, National Institute of Mental Health, National Institute on Drug Abuse, National Institute of Nursing Research, National Cancer Institute, National Institute on Alcohol Abuse and Alcoholism, National Institute on Deafness and Other Communication Disorders, and National Institute of Diabetes and Digestive and Kidney Diseases. MWCCS data collection is also supported by the University of California San Francisco Clinical and Translational Science Institute (UL1-TR000004), Atlanta Center for AIDS Research (P30-AI-050409), University of North Carolina Center for AIDS Research (P30-AI-050410), and University of Alabama at Birmingham Center for AIDS Research (P30-AI-027767).

***Disclaimer.*** The contents of this publication are solely the responsibility of the authors and do not represent the official views of the National Institutes of Health.

***Patient consent statement.*** WIHS participants provided written informed consent, and participating sites received institutional review board approval before enrolling participants. This analysis received approval by the MWCCS executive committee and University of Alabama at Birmingham institutional review board.

***Financial support*.** This work was supported by the MWCCS to the principal investigators of the Atlanta clinical research site (U01-HL146241 to I. O.), data analysis and coordination center (U01-HL146193 to S. J. G.), Miami clinical research site (U01-HL146203 to M. F.), University of Alabama at Birmingham–Mississippi clinical research site (U01-HL146192 to M.-C. K.), and University of North Carolina clinical research site (U01-HL146194 to A. A. A.); and by the Emory Specialized Center of Research Excellence on Sex Differences (U54AG062334 to I. O.).
