# Supplementary material for: Disparities in Hypertension Prevalence, Awareness, Treatment, and Control Among Women Living With and Without HIV in the US South
Source: Open Forum Infect Dis. 2023 Dec 18;11(1):ofad642. doi: 10.1093/ofid/ofad642 (PMC10776242; doi:10.1093/ofid/ofad642)
Supplement: ofad642_Supplementary_Data [file ofad642_supplementary_data.doc]

Supplemental Figure 1. Proportions of hypertension outcomes by race/ethnicity for women enrolled at Southern sites of Women’s Interagency HIV Study (WIHS) (N=723)


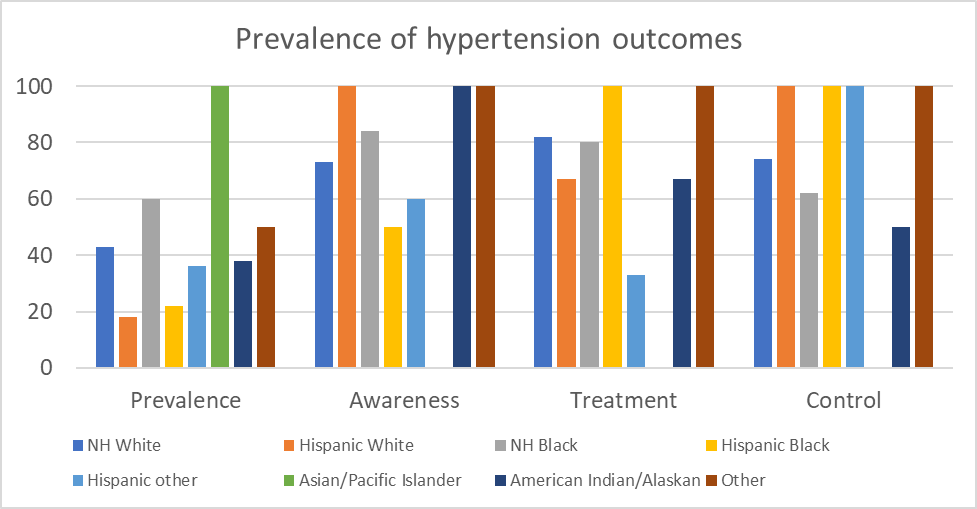


|  | Prevalence | Awareness | Treatment | Control |
| --- | --- | --- | --- | --- |
| NH White | 30/70 | 22/30 | 19/22 | 14/19 |
| Hispanic White | 3/17 | 3/3 | 2/3 | 2/2 |
| NH Black | 361/602 | 302/361 | 252/302 | 156/252 |
| Hispanic Black | 2/9 | 1/2 | 1/1 | 1/1 |
| Hispanic other | 5/14 | 3/5 | 1/3 | 1/1 |
| Asian/Pacific Islander | 1/1 | 0/1 | NA | NA |
| American Indian/Alaskan | 3/8 | 3/3 | 2/3 | 1/2 |
| Other | 1/2 | 1/1 | 1/1 | 1/1 |

Supplemental Figure 2. Flowchart of exclusion criteria for women enrolled at Southern sites of the Women’s Interagency HIV Study (WIHS)

* Other includes Asian/Pacific Islander, American Indian/Alaskan, and other


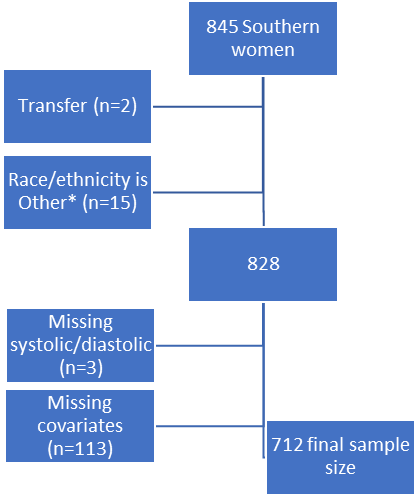


Supplemental Table 1. Characteristics of study population for women enrolled at Southern sites of the Women’s Interagency HIV Study (WIHS) included and excluded from analyses

|  | Included (N=712) | Excluded (N=133) |
| --- | --- | --- |
| Baseline age, mean (SD) | 43.2 (9.4) | 42.7 (9.2) |
| Race/ethnicity, %  NH Black  NH White  Hispanic | 602 (84.6)  70 (9.8)  40 (5.6) | 98 (73.7)  9 (6.8)  11 (8.3) |
| Education, %  Less than HS  HS graduate  Some college  College graduate or higher | 214 (30.1)  222 (31.2)  226 (31.7)  50 (7.0) | 41 (30.8)  44 (33.1)  35 (26.3)  13 (9.8) |
| Health insurance, %  Uninsured  Medicaid only  ADAP only  Other* | 166 (23.3)  261 (36.7)  107 (15.0)  178 (25.0) | 41 (31.1)  34 (25.8)  29 (22.0)  28 (21.2) |
| Smoking status, %  Never  Former  Current | 289 (40.6)  93 (13.1)  330 (46.4) | 50 (37.6)  17 (12.8)  66 (49.6) |
| Alcohol use†, %  None  Moderate  Heavy | 336 (47.2)  251 (35.3)  125 (17.6) | 51 (38.6)  49 (37.1)  32 (24.2) |
| Substance use, %  None  Marijuana only  Non-IV drug use  IV drug use | 233 (32.7)  117 (16.4)  316 (44.4)  46 (6.5) | 43 (32.3)  28 (21.1)  48 (36.1)  14 (10.5) |
| BMI‡, %  Underweight/Normal  Overweight  Obese | 123 (17.3)  163 (22.9)  426 (59.8) | 36 (28.6)  34 (27.0)  56 (44.4) |
| History of CVD§, % | 64 (9.0) | 16 (12.0) |
| Diabetes ||, % | 84 (11.8) | 10 (7.5) |
| eGFR, mean (SD) | 100.8 (23.7) | 103.8 (22.4) |
| FIB-4, mean (SD) | 1.1 (1.0) | 1.6 (2.0) |
| APRI, mean (SD) | 0.3 (0.4) | 0.4 (0.8) |
| Hepatitis C, % | 84 (11.8) | 23 (17.6) |
| Depressive symptoms #, % | 327 (45.9) | 55 (43.7) |
| HIV status, %  Negative  Suppressed**  Unsuppressed | 219 (30.8)  363 (51.0)  130 (18.3) | 16 (12.0)  26 (19.6)  91 (68.4) |
| Current ART usage, %  None  INSTIs  Non-INSTIs | 28 (5.7)  162 (32.9)  303 (61.5) | 91 (77.8)  4 (3.4)  22 (18.8) |
| Duration of ART (years), mean (SD) | 4.1 (2.7) | 4.6 (3.0) |
| CD4 count (cells/uL), mean (SD) | 752.5 (414.6) | 555.5 (334.6) |
| AIDS diagnosis, % | 54 (7.6) | 8 (6.0) |

NH: non-Hispanic; HS: high school; ADAP: AIDS Drug Assistance Program; IV: intravenous; CVD: cardiovascular disease; eGFR: estimated glomerular filtration rate; FIB-4: hepatic fibrosis; APRI: aspartate aminotransferase/platelet ratio; ART: antiretroviral therapy; INSTI: integrase inhibitors; AIDS: acquired immunodeficiency syndrome

* Other includes private, Medicare, combination of insurances, and other insurance

† Rank based on none, moderate (1-7 drinks/week), and heavy (>7 drinks/week) according to NIAAA guidelines for women

‡ BMI defined as underweight/normal (<25 kg/m^2^), overweight (25 to <30 kg/m^2^), and obese (≥30 kg/m^2^)

§ History of CVD includes, MI, hospitalization for CHF, stroke, TIA, hospitalization for angina, or surgery on heart vessels

|| Diabetes defined as fasting glucose ≥126 mg/dL, HgbA1C ≥6.5%, confirmed self-report diagnosis, or ever self-reported anti-diabetic medication

# Depressive symptoms defined CES-D score ≥16

** Cut off for viral suppression was <20 copies/ml

Supplemental Table 2. Unadjusted and adjusted prevalence ratios and 95% confidence intervals (CI) for the association between hypertension (≥ 130/80 mmHg) outcomes and race/ethnicity for women enrolled at Southern sites of the Women’s Interagency HIV Study (WIHS)

| Prevalence ratio (95% CI) | | | | |
| --- | --- | --- | --- | --- |
|  | *Model 1*  *Race/ethnicity only*  *(unadjusted)* | *p-value*† | *Model 2*  *Model 1 + all covariates*  *(fully adjusted)* | *p-value*† |
| Prevalence (N=712) | | | | |
| Race/ethnicity  NH Black  NH White  Hispanic | Ref.  0.80 (0.64 – 1.00)  0.58 (0.40 – 0.86) | **.0010** | Ref.  0.80 (0.65 – 0.99)  0.70 (0.49 – 1.01) | **.0114** |
| Awareness (N=464) | | | | |
| Race/ethnicity  NH Black  NH White  Hispanic | Ref.  0.79 (0.60 – 1.04)  0.59 (0.34 – 1.04) | **.0244** | Ref.  0.73 (0.56 – 0.94)  0.62 (0.37 – 1.04) | **.0053** |
| Treatment (N=331) | | | | |
| Race/ethnicity  NH Black  NH White  Hispanic | Ref.  1.02 (0.83 – 1.25)  0.71 (0.37 – 1.36) | .5253 | Ref.  0.95 (0.79 – 1.16)  0.72 (0.43 – 1.19) | .3477 |
| Control (N=275) | | | | |
| Race/ethnicity  NH Black  NH White  Hispanic | Ref.  0.82 (0.42 – 1.62)  1.95 (1.08 – 3.50) | .3546 | Ref.  0.72 (0.34 – 1.54)  2.94 (1.34 – 6.43) | .1697 |

NH: non-Hispanic

† Type 3 significant p-value indicated that at least one group is different than another

Supplemental Table 3. Unadjusted and adjusted prevalence ratios and 95% confidence intervals (CI) for the association between hypertension outcomes and race/ethnicity with imputed values for women enrolled at Southern sites of the Women’s Interagency HIV Study (WIHS)

| Prevalence ratio (95% CI) | | | | |
| --- | --- | --- | --- | --- |
|  | *Model 1*  *Race/ethnicity only*  *(unadjusted)* | *p-value*† | *Model 2*  *Model 1 + all covariates*  *(fully adjusted)* | *p-value*† |
| Prevalence (N=825) | | | | |
| Race/ethnicity  NH Black  NH White  Hispanic | Ref.  0.72 (0.56 – 0.94)  0.46 (0.29 – 0.72) | **<.0001** | Ref.  0.69 (0.54 – 0.89)  0.56 (0.37 – 0.87) | **.0001** |
| Awareness (N=461) | | | | |
| Race/ethnicity  NH Black  NH White  Hispanic | Ref.  0.89 (0.72 – 1.09)  0.95 (0.72 – 1.25) | .4764 | Ref.  0.85 (0.70 – 1.04)  0.88 (0.67 – 1.16) | .1890 |
| Treatment (N=378) | | | | |
| Race/ethnicity  NH Black  NH White  Hispanic | Ref.  0.96 (0.77 – 1.21)  0.81 (0.51 – 1.26) | .5807 | Ref.  0.96 (0.80 – 1.15)  0.67 (0.41 – 1.08) | .2425 |
| Control (N=308) | | | | |
| Race/ethnicity  NH Black  NH White  Hispanic | Ref.  1.21 (0.93 – 1.59)  1.15 (0.72 – 1.86) | .4071 | Ref.  1.22 (0.90 – 1.65)  1.58 (0.90 – 2.76) | .1625 |

NH: non-Hispanic

† Type 3 significant p-value indicated that at least one group is different than another

Supplemental Table 4. Unadjusted and adjusted prevalence ratios and 95% confidence intervals (CI) for the association between hypertension outcomes and race/ethnicity defined as Black and non-Black for women enrolled at Southern sites of the Women’s Interagency HIV Study (WIHS)

| Prevalence ratio (95% CI) | | | | |
| --- | --- | --- | --- | --- |
|  | *Model 1*  *Race/ethnicity only*  *(unadjusted)* | *p-value* | *Model 2*  *Model 1 + all covariates*  *(fully adjusted)* | *p-value* |
| Prevalence (N=723) | | | | |
| Race/ethnicity  Black  Non-Black | Ref.  0.65 (0.51 – 0.82) | **<.0001** | Ref.  0.67 (0.54 – 0.84) | **.0001** |
| Awareness (N=406) | | | | |
| Race/ethnicity  Black  Non-Black | Ref.  0.89 (0.74 – 1.07) | .1987 | Ref.  0.86 (0.73 – 1.02) | .0665 |
| Treatment (N=335) | | | | |
| Race/ethnicity  Black  Non-Black | Ref.  0.94 (0.76 – 1.15) | .5176 | Ref.  0.89 (0.73 – 1.07) | .2048 |
| Control (N=278) | | | | |
| Race/ethnicity  Black  Non-Black | Ref.  1.22 (0.96 – 1.56) | .1387 | Ref.  1.27 (0.96 – 1.67) | .1201 |

Supplemental Table 5. Unadjusted and adjusted prevalence ratios and 95% confidence intervals (CI) for the association between hypertension outcomes and race/ethnicity among women with HIV enrolled at Southern sites of the Women’s Interagency HIV Study (WIHS)

| Prevalence ratio (95% CI) | | | | |
| --- | --- | --- | --- | --- |
|  | *Model 1*  *Race/ethnicity only*  *(unadjusted)* | *p-value*† | *Model 2*  *Model 1 + all covariates*  *(fully adjusted)* | *p-value*† |
| Prevalence (N=493) | | | | |
| Race/ethnicity  NH Black  NH White  Hispanic | Ref.  0.80 (0.60 – 1.08)  0.42 (0.22 – 0.81) | **.0018** | Ref.  0.75 (0.56 – 0.99)  0.56 (0.32 – 0.98) | **.0058** |
| Awareness (N=286) | | | | |
| Race/ethnicity  NH Black  NH White  Hispanic | Ref.  0.84 (0.65 – 1.09)  0.85 (0.53 – 1.36) | .3108 | Ref.  0.82 (0.61 – 1.03)  0.82 (0.56 – 1.22) | .1105 |
| Treatment (N=237) | | | | |
| Race/ethnicity  NH Black  NH White  Hispanic | Ref.  0.97 (0.78 – 1.22)  0.71 (0.35 – 1.45) | .5895 | Ref.  0.95 (0.77 – 1.17)  0.70 (0.40 – 1.23) | .3756 |
| Control (N=208) | | | | |
| Race/ethnicity  NH Black  NH White  Hispanic | Ref.  1.04 (0.71 – 1.51)  1.56 (1.40 – 1.73) | .2184 | Ref.  1.01 (0.68 – 1.49)  2.05 (1.37 – 3.06) | .1995 |

NH: non-Hispanic

† Type 3 significant p-value indicated that at least one group is different than another

Supplemental Table 6. Unadjusted and adjusted prevalence ratios and 95% confidence intervals (CI) for the association between hypertension outcomes and HIV status for women enrolled at Southern sites of the Women’s Interagency HIV Study (WIHS)

| Prevalence ratio (95% CI) | | | | |
| --- | --- | --- | --- | --- |
|  | *Model 1*  *HIV status only*  *(unadjusted)* | *p-value*† | *Model 2*  *Model 1 + all covariates*  *(fully adjusted)** | *p-value*† |
| Prevalence (N=712) | | | | |
| HIV status  Suppressed  Unsuppressed  Negative | Ref.  0.97 (0.82 – 1.16)  0.90 (0.77 – 1.05) | .3806 | Ref.  0.99 (0.84 – 1.16)  0.85 (0.71 – 1.01) | .1679 |
| Awareness (N=401) | | | | |
| HIV status  Suppressed  Unsuppressed  Negative | Ref.  0.99 (0.88 – 1.12)  0.98 (0.89 – 1.09) | .9587 | Ref.  1.00 (0.88 – 1.13)  0.95 (0.84 – 1.09) | .7852 |
| Treatment (N=331) | | | | |
| HIV status  Suppressed  Unsuppressed  Negative | Ref.  0.92 (0.79 – 1.06)  0.81 (0.70 – 0.93) | **.0098** | Ref.  0.96 (0.84 – 1.11)  0.83 (0.71 – 0.98) | .0824 |
| Control (N=275) | | | | |
| HIV status  Suppressed  Unsuppressed  Negative | Ref.  0.77 (0.58 – 1.03)  0.85 (0.68 – 1.07) | .0966 | Ref.  0.76 (0.57 – 1.02)  0.87 (0.66 – 1.16) | .1073 |

* Fully adjusted Poisson model does not have duration of ART, AIDS diagnosis, or current ART usage

† Type 3 significant p-value indicated that at least one group is different than another

Supplemental Table 7. Unadjusted and adjusted prevalence ratios and 95% confidence intervals (CI) for the association between hypertension outcomes and HIV with imputed values for women enrolled at Southern sites of the Women’s Interagency HIV Study (WIHS)

| Prevalence ratio (95% CI) | | | | |
| --- | --- | --- | --- | --- |
|  | *Model 1*  *HIV status only*  *(unadjusted)* | *p-value* | *Model 2*  *Model 1 + all covariates*  *(fully adjusted)** | *p-value* |
| Prevalence (N=825) | | | | |
| HIV status  WLWOH  WLWH | Ref.  1.08 (0.94 – 1.24) | .2778 | Ref.  1.12 (0.96 – 1.32) | .1545 |
| Awareness (N=461) | | | | |
| HIV status  WLWOH  WLWH | Ref.  1.02 (0.81 – 1.28) | .7413 | Ref.  1.03 (0.92 – 1.19) | .5019 |
| Treatment (N=378) | | | | |
| HIV status  WLWOH  WLWH | Ref.  1.22 (1.05 – 1.41) | **.0060** | Ref.  1.20 (1.02 – 1.41) | **.0330** |
| Control (N=308) | | | | |
| HIV status  WLWOH  WLWH | Ref.  1.13 (0.85 – 1.41) | .2863 | Ref.  1.13 (0.86 – 1.49) | .3788 |

WLWOH: women living without HIV; WLWH: women living with HIV

* Fully adjusted Poisson model does not have duration of ART, AIDS diagnosis, or current ART usage

Supplemental Table 8. Unadjusted and adjusted prevalence ratios and 95% confidence intervals (CI) for the association between hypertension (≥ 130/80 mmHg) outcomes and HIV for women enrolled at Southern sites of the Women’s Interagency HIV Study (WIHS)

| Prevalence ratio (95% CI) | | | | |
| --- | --- | --- | --- | --- |
|  | *Model 1*  *HIV status only*  *(unadjusted)* | *p-value* | *Model 2*  *Model 1 + all covariates*  *(fully adjusted)** | *p-value* |
| Prevalence (N=712) | | | | |
| HIV status  WLWOH  WLWH | Ref.  1.09 (0.97 – 1.24) | .1430 | Ref.  1.14 (0.98 – 1.32) | .0778 |
| Awareness (N=464) | | | | |
| HIV status  WLWOH  WLWH | Ref.  1.02 (0.90 – 1.17) | .7206 | Ref.  1.08 (0.92 – 1.27) | .3563 |
| Treatment (N=331) | | | | |
| HIV status  WLWOH  WLWH | Ref.  1.21 (1.05 – 1.49) | **.0065** | Ref.  1.19 (1.01 – 1.40) | **.0353** |
| Control (N=275) | | | | |
| HIV status  WLWOH  WLWH | Ref.  1.16 (0.80 – 1.68) | .4082 | Ref.  1.12 (0.73 – 1.72) | .6085 |

WLWOH: women living without HIV; WLWH: women living with HIV

* Fully adjusted Poisson model does not have duration of ART, AIDS diagnosis, or current ART usage

Supplemental Table 9. Associations of characteristics of study population for women enrolled at Southern sites of the Women’s Interagency HIV Study (WIHS) by hypertension outcomes

|  | Presence hypertension (N=401) | Absence hypertension (N=311) | p-value | Presence awareness (N=331) | Absence awareness (N=70) | p-value | Presence treatment (N=264) | Absence treatment (N=67) | p-value | Presence control (N=165) | Absence control (N=99) | p-value |
| --- | --- | --- | --- | --- | --- | --- | --- | --- | --- | --- | --- | --- |
| Baseline age, mean (SD) | 46.2 (9.0) | 39.4 (8.5) | **<.0001** | 46.4 (8.8) | 45.2 (9.7) | .3119 | 47.9 (8.0) | 40.4 (9.4) | **<.0001** | 47.8 (7.9) | 48.1 (8.2) | .7920 |
| Race/ethnicity, %  NH Black  NH White  Hispanic | 361 (90.0)  30 (7.5)  10 (2.5) | 241 (77.5)  40 (12.9)  30 (9.7) | **<.0001** | 302 (91.2)  22 (6.7)  7 (2.11) | 59 (84.3)  8 (11.4)  3 (4.3) | .2051 | 242 (91.7)  18 (6.8)  4 (1.5) | 60 (89.6)  4 (6.0)  3 (4.5) | .2872 | 148 (89.7)  13 (7.9)  4 (2.4) | 94 (95.0)  5 (5.1)  0 (0.0) | .2586 |
| Education, %  Less than HS  HS graduate  Some college  College graduate or higher | 121 (30.2)  123 (30.7)  125 (31.2)  32 (8.0) | 93 (29.9)  99 (31.8)  101 (32.5)  18 (5.8) | .7120 | 104 (31.4)  98 (29.6)  102 (30.8)  27 (8.2) | 17 (24.3)  25 (35.7)  23 (32.9)  5 (7.1) | .6087 | 79 (29.9)  75 (28.4)  89 (33.7)  21 (8.0) | 25 (37.3)  23 (34.3)  13 (19.4)  6 (9.0) | .1593 | 46 (27.9)  50 (30.3)  56 (33.9)  13 (7.9) | 33 (33.3)  25 (25.3)  33 (33.3)  8 (8.1) | .7576 |
| Health insurance, %  Uninsured  Medicaid only  ADAP only  Other* | 94 (23.4)  142 (35.4)  52 (13.0)  113 (28.2) | 72 (23.2)  119 (38.3)  55 (17.7)  65 (20.9) | .0821 | 78 (23.6)  118 (35.7)  41 (12.4)  94 (28.4) | 16 (22.9)  24 (34.3)  11 (15.7)  19 (27.1) | .9036 | 53 (20.1)  94 (35.6)  32 (12.1)  85 (32.2) | 25 (37.3)  24 (35.8)  9 (13.4)  9 (13.4) | **.0038** | 32 (19.4)  58 (35.2)  21 (12.7)  54 (32.7) | 21 (21.2)  36 (36.4)  11 (11.1)  31 (31.3) | .9602 |
| Smoking status, %  Never  Former  Current | 148 (36.9)  61 (15.2)  192 (47.9) | 141 (45.3)  32 (10.3)  138 (44.4) | **.0337** | 116 (35.1)  53 (16.0)  162 (48.9) | 32 (45.7)  8 (11.4)  30 (42.9) | .2202 | 93 (35.2)  44 (16.7)  127 (48.1) | 23 (34.3)  9(13.4)  35 (52.2) | .7604 | 63 (38.2)  31 (18.8)  71 (43.0) | 30 (30.3)  13 (13.1)  56 (56.6) | .0985 |
| Alcohol use†, %  None  Moderate  Heavy | 191 (47.6)  135 (33.7)  75 (18.7) | 145 (46.6)  116 (37.3)  50 (16.1) | .5012 | 156 (47.1)  110 (33.2)  65 (19.6) | 35 (50.0)  25 (35.7)  10 (14.3) | .5797 | 128 (48.5)  92 (34.9)  44 (16.7) | 28 (41.8)  18 (26.9)  21 (31.3) | **.0248** | 81 (49.1)  58 (35.2)  26 (15.8) | 47 (47.5)  34 (34.3)  18 (18.2) | .8767 |
| Substance use, %  None  Marijuana only  Non-intravenous drug use  Intravenous drug use | 119 (29.7)  54 (13.5)  199 (49.6)  29 (7.2) | 114 (36.7)  63 (20.3)  117 (37.6)  17 (5.5) | **.0028** | 98 (29.6)  39 (11.8)  169 (51.1)  25 (7.6) | 21 (30.0)  15 (21.4)  30 (42.9)  4 (5.7) | .1683 | 83 (31.4)  28 (10.6)  133 (50.4)  20 (7.6) | 11 (16.4)  11 (16.4)  36 (53.7)  5 (7.5) | .3721 | 57 (34.6)  18 (10.9)  79 (47.9)  11 (6.7) | 26 (26.3)  10 (10.1)  54 (54.6)  9 (9.1) | .4910 |
| BMI‡, %  Underweight/Normal  Overweight  Obese | 57 (14.2)  73 (18.2)  271 (67.6) | 66 (21.2)  90 (28.9)  155 (49.8) | **<.0001** | 46 (13.9)  58 (17.5)  227 (68.6) | 11 (15.7)  15 (21.4)  44 (62.9) | .6381 | 31 (11.7)  48 (18.2)  185 (70.1) | 15 (22.4)  10 (14.9)  42 (62.7) | .0778 | 16 (9.7)  35 (21.2)  114 (69.1) | 15 (15.2)  13 (13.1)  71 (71.7) | .1458 |
| History of CVD§, % | 50 (12.5) | 14 (4.5) | **.0002** | 49 (14.8) | 1 (1.43) | **.0021** | 46 (17.4) | 3 (4.5) | **.0077** | 24 (14.6) | 22 (22.2) | .1114 |
| Diabetes ||, % | 72 (18.0) | 12 (3.9) | **<.0001** | 62 (18.7) | 10 (14.3) | .3786 | 54 (20.5) | 8 (11.9) | .1107 | 36 (21.8) | 18 (18.2) | .4782 |
| eGFR, mean (SD) | 97.9 (25.5) | 104.5 (20.7) | **.0001** | 96.0 (26.1) | 106.9 (20.3) | **.0002** | 93.3 (25.7) | 106.5 (24.9) | **.0002** | 94.0 (23.9) | 92.2 (28.6) | .5927 |
| FIB-4, mean (SD) | 1.1 (1.1) | 1.0 (0.6) | **.0172** | 1.1 (1.2) | 1.1 (0.8) | .7297 | 1.1 (0.9) | 1.1 (1.9) | .9830 | 1.1 (1.0) | 1.2 (0.8) | .2781 |
| APRI, mean (SD) | 0.3 (0.5) | 0.3 (0.2) | .1533 | 0.3 (0.5) | 0.3 (0.4) | .6434 | 0.3 (0.3) | 0.4 (0.9) | .5275 | 0.3 (0.3) | 0.3 (0.3) | .1816 |
| Hepatitis C, % | 59 (14.7) | 25 (8.0) | **.0062** | 46 (13.9) | 13 (18.6) | .3159 | 37 (14.0) | 9 (13.4) | .9021 | 20 (12.1) | 17 (17.2) | .2525 |
| Depressive symptoms #, % | 191 (47.6) | 136 (43.7) | .3002 | 165 (49.9) | 26 (37.1) | .0531 | 127 (48.1) | 38 (56.7) | .2081 | 77 (46.7) | 50 (50.5) | .5456 |
| HIV status, %  Negative  Unsuppressed  Suppressed** | 115 (28.7)  74 (18.5)  212 (52.9) | 104 (33.4)  56 (18.0)  151 (48.6) | .3771 | 94 (28.4)  61 (18.4)  176 (53.2) | 21 (30.0)  13 (18.6)  36 (51.4) | .9581 | 65 (24.6)  48 (18.2)  151 (57.2) | 29 (43.3)  13 (19.4)  25 (37.3) | **.0051** | 37 (22.4)  25 (15.2)  103 (62.4) | 28 (28.3)  23 (23.2)  48 (48.5) | .0748 |
| Current ART usage, %  None  INSTIs  Non-INSTIs | 13 (4.6)  92 (32.2)  181 (63.3) | 15 (7.3)  70 (33.8)  122 (58.9) | .3658 | 12 (5.1)  77 (32.5)  148 (62.5) | 1 (2.0)  15 (30.6)  33 (67.4) | .6021 | 10 (5.0)  66 (33.2)  123 (61.8) | 2 (5.3)  11 (29.0)  25 (65.8) | .8784 | 5 (3.9)  41 (32.0)  82 (64.1) | 5 (7.0)  25 (35.2)  41 (57.8) | .5149 |
| Duration of ART (years), mean (SD) | 4.1 (2.7) | 3.9 (2.7) | .4121 | 4.2 (2.8) | 3.7 (2.6) | .2301 | 4.4 (2.8) | 3.5 (2.3) | .0592 | 4.3 (2.9) | 4.5 (2.6) | .5103 |
| CD4 count (cells/uL), mean (SD) | 751.5 (419.8) | 753.9 (408.4) | .9380 | 748.5 (425.4) | 765.6 (394.8) | .7568 | 750.7 (433.4) | 739.6 (395.6) | .8496 | 753.1 (412.1) | 746.7 (468.8) | .9071 |
| AIDS diagnosis, % | 33 (8.2) | 21(6.8) | .3496 | 27 (8.2) | 6 (8.6) | .9508 | 26 (9.9) | 1 (1.5) | **.0025** | 17 (10.3) | 9 (9.1) | .5603 |

NH: non-Hispanic; HS: high school; ADAP: AIDS Drug Assistance Program; IV: intravenous; CVD: cardiovascular disease; eGFR: estimated glomerular filtration rate; FIB-4: hepatic fibrosis; APRI: aspartate aminotransferase/platelet ratio; ART: antiretroviral therapy; INSTI: integrase inhibitors; AIDS: acquired immunodeficiency syndrome

* Other includes private, Medicare, combination of insurances, and other insurance

† Rank based on none, moderate (1-7 drinks/week), and heavy (>7 drinks/week) according to NIAAA guidelines for women

‡ BMI defined as underweight/normal (<25 kg/m^2^), overweight (25 to <30 kg/m^2^), and obese (≥30 kg/m^2^)

§ History of CVD includes, MI, hospitalization for CHF, stroke, TIA, hospitalization for angina, or surgery on heart vessels

|| Diabetes defined as fasting glucose ≥126 mg/dL, HgbA1C ≥6.5%, confirmed self-report diagnosis, or ever self-reported anti-diabetic medication

# Depressive symptoms defined CES-D score ≥16

** Cut off for viral suppression was <20 copies/ml
